# Comments on “A rare case of biatrial myxoma in an 11-year-old girl patient with thromboembolic stroke: A case report”

**DOI:** 10.1016/j.ijscr.2025.111756

**Published:** 2025-07-30

**Authors:** Esmael Amirazodi, Fatemeh Tahmasebi, Arman Abroumand Gholami

**Affiliations:** a Department of Neurology, Ahvaz Jundishapur University of Medical sciences, Ahvaz, Iran; b Department of Anatomy, Faculty of Medicine, Saveh University of Medical Sciences, Saveh, Iran; c Department of Neuroscience, Faculty of Medicine, Mashhad University of Medical Sciences, Mashhad, Iran; d Nervous System Stem Cell Research Center, Semnan University of Medical Sciences, Semnan, Iran

Dear Editor,

We read with great interest the case report by Laksmono et al., describing a rare presentation of biatrial myxoma in an 11-year-old girl with thromboembolic stroke [1]. This case holds substantial educational value by drawing attention to an uncommon cardiac pathology in the pediatric population with acute neurological manifestations. While atrial myxomas are well recognized in adults, their occurrence in children particularly with biatrial involvement is exceedingly rare and warrants heightened clinical suspicion. This report prompts reflection on several key clinical and diagnostic considerations.

First, the diagnostic trajectory merits further emphasis. Although the authors rightly highlight the utility of transthoracic echocardiography (TTE) for identifying intracardiac masses, the early use of transesophageal echocardiography (TEE) could have enhanced spatial resolution and facilitated prompt preoperative planning, even in pediatric settings. Given the embolic event, cardiac imaging should have been pursued immediately following the neurologic presentation to minimize diagnostic delays—an essential learning point for clinicians [2]. Furthermore, while the report documents successful surgical excision, it lacks detail regarding intraoperative findings, cardiopulmonary bypass strategies, and any perioperative complications. Surgical management of pediatric intracardiac tumors, especially with biatrial extension, presents unique challenges, including the need to balance complete tumor resection with the preservation of valvular and septal integrity. A more detailed surgical description would have strengthened the report's practical utility for cardiothoracic surgeons.

Second, this case reinforces the potential of cardiac myxomas as underrecognized sources of cardioembolic strokes in children. While infective endocarditis and congenital heart anomalies are more commonly implicated, this case underscores the necessity of including benign cardiac tumors in the differential diagnosis for pediatric patients presenting with ischemic stroke of unclear etiology. Incorporating early cardiac imaging into pediatric stroke workups, particularly in the absence of conventional vascular risk factors, may help reduce diagnostic errors and delays.

Third, the report would benefit from greater diagnostic specificity regarding imaging modalities. Although TTE confirmed the presence of a mass, it remains unclear whether TEE or cardiac magnetic resonance imaging (MRI) was utilized to better define the anatomical characteristics of the tumor. In cases involving biatrial tumors, multimodal imaging is crucial for delineating tumor extension, assessing valvular involvement, and evaluating embolic potential [3]. The omission of these details limits the case's educational impact and the broader applicability of its surgical insights.

Fourth, the surgical approach was commendable. The successful resection of the biatrial tumors with preservation of cardiac function illustrates the importance of surgical preparedness and the necessity for intraoperative flexibility, given the anatomical unpredictability of such tumors [4]. Nonetheless, elaboration on perioperative care and post-resection follow-up would have been valuable especially considering recurrence risks in familial cases.

Finally, the potential for underlying genetic syndromes should not be overlooked. Although this case was characterized as sporadic, it is prudent to consider screening for Carney complex or other familial conditions when multiple cardiac myxomas are present. Genetic counseling and family screening in such scenarios may be life-saving [5].

In conclusion, this case underscores a critical clinical lesson: though biatrial myxomas in children are rare, they carry significant embolic risk and require prompt diagnosis and timely surgical intervention. This report reinforces the importance of interdisciplinary collaboration among neurologists, cardiologists, and cardiothoracic surgeons in managing pediatric strokes with atypical etiologies. We commend the authors for contributing to the literature on this rare clinical entity and encourage further multi-institutional data collection to better define diagnostic pathways and management strategies in such cases.

## CRediT authorship contribution statement

Esmael Amirazodi: Writing and editing the draft.
Fatemeh Tahmasebi: Writing and editing the draft.
Arman Abroumand Gholami: Study design, data collection, writing and editing the draft.

All authors read and approved the final version of the manuscript.

## Ethical approval

Not applicable for this study.

## Guarantor

All the authors of this paper accept full responsibility for the work and/or the conduct of the study, had access to the data, and controlled the decision to publish.

## Research registration number

1. Name of the registry: Not applicable.
2. Unique Identifying number or registration ID: Not applicable.
3. Hyperlink to your specific registration: Not applicable.

## Funding

None.

## Declaration of competing interest

The authors have no conflict of interest.
